# Conceptualization and Preliminary Characterization of Poloxamer-Based Hydrogels for Biomedical Applications

**DOI:** 10.1021/acs.bioconjchem.5c00030

**Published:** 2025-05-19

**Authors:** Marta Tuszynska, Joanna Skopinska-Wisniewska, Mateusz Bartniak, Anna Bajek

**Affiliations:** † Nicolaus Copernicus University in Torun, Ludwik Rydygier Collegium Medicum in Bydgoszcz, Faculty of Oncology, Lukasiewicza 1 St., Bydgoszcz 85-094, Poland; ‡ Faculty of Chemistry, 49577 Nicolaus Copernicus University in Torun, Gagarina 7 St., Torun 87-100, Poland; § Faculty of Mechanical Engineering, 318880 Institute of Materials Science and Engineering, Lodz University of Technology, 1/15 Stefanowskiego St., Lodz 90-924, Poland

## Abstract

Poloxamers are widely used in biomedical applications, but their effectiveness depends on achieving an optimal sol–gel phase transition near body temperature. This study evaluates three different poloxamer mixtures for their potential in treating meniscus tears, focusing on gel formation, injectability, and cell compatibility. The rheological properties, cytotoxicity assessments, and cellular migration experiments were studied using NIH/3T3 fibroblast cells as the standard experimental model for primary research. The poloxamer hydrogels showed properties well suited to injectable or drug delivery systems. Specifically, the combination of Synperonic F108 and Poloxamer 188 tended to show less adhesion and more aggregation, followed by a greater number of viable cells, suggesting its utility as a coating or foundational matrix. Concurrently, the Kolliphor 407 and Poloxamer 188 combination exhibited increased viscosity, maintaining a gel state at physiological temperature. Its biocompatibility indicated the potential for injectable controlled-release systems for musculoskeletal injuries. Our findings demonstrate that the poloxamer concentration and composition significantly influence their biomedical applications. These triblock copolymer systems indicated useful characteristics for surgical applications, such as favorable sol–gel transition kinetics and biocompatibility, suggesting potential applications in osteoarticular regeneration.

## Graphical abstract

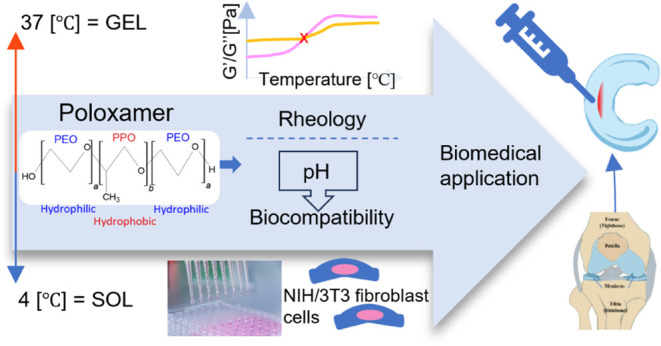

## 1 Introduction

In recent years, the field of biomaterials has significantly impacted biomedical engineering. Currently, researchers are working on biocompatible, nontoxic materials that are easy to prepare. These materials must fulfill the requirements in the growing field of tissue engineering. Among these biomaterials, poloxamer-based hydrogels have gained interest as a promising material class due to their unique physicochemical properties and adaptability in various temperatures and conditions.^1^ Although extensively studied for various applications, their potential as injectable systems specifically for meniscus tear regeneration remains insufficiently explored, creating a significant research gap that this study aims to address. Poloxamers, mostly known by their brand name Pluronics (manufactured by BASF, Ludwigshafen, Germany), are a family of triblock copolymers with a central hydrophobic poly­(propylene oxide) (PPO) segment flanked by two hydrophilic poly­(ethylene oxide) (PEO) blocks. They exhibit the general formula (PEO)­x-(PPO)­y-(PEO)­x. The different types of poloxamers are characterized by their molecular weight, PEO/PPO ratio, and specific grade. Their amphiphilic nature allows them to form thermoresponsive hydrogels that exhibit reversible sol–gel transitions with an increase in temperature. This characteristic makes them particularly valuable in biomedical applications where controlled delivery and site-specific release of therapeutic agents are essential.^2–4^ Recent advances in injectable biomaterials have shown promising results for targeted drug delivery and tissue regeneration.^5–11^ It was demonstrated that novel nanocomposite hydrogels can provide sustained release of growth factors in cartilage repair and highlighted the importance of hydrogel formulation on cellular response and tissue integration in minimally invasive approaches.^7,11–14^ The ability to precisely target the injured area and the minimally invasive nature of the injection make these hydrogels an attractive option for improving recovery outcomes and patient comfort.^15–17^

While injectable hydrogels have been studied for cartilage, bone, and osteochondral defects, poloxamer-based hydrogels used in meniscus tissue engineering are still underexplored. For minor defects, injectable hydrogels offer shape conformity, eliminate the need for suturing, and allow for the delivery of growth factors and cells. However, many current hydrogels lack mechanical stability.^18,19^ Synthetic polymer-based hydrogels, known for their mechanical and biological properties in 3D-printed constructs, could be promising for injectable applications in meniscus repair.^20^ −.^21,22^

The biocompatibility of poloxamers makes them suitable for various biomedical and pharmaceutical applications. The selected poloxamer combinations were based on previous literature studies, indicating their potential for gelation at body temperature and favorable rheological properties. The combinations of Kolliphor K407/Poloxamer 188, Synperonic F108/Kolliphor K407, and Synperonic F108/Poloxamer 188 were chosen due to their complementary properties, and the weight ratio of 10–30% was selected based on preliminary studies showing that lower concentrations do not form stable gels, while higher concentrations cause excessive viscosity at room temperature, making injection difficult. It is important to note that while these nonionic surfactants are generally considered biocompatible, individual formulations and concentrations can affect their biocompatibility in specific applications. Additionally, the molecular weight, PEO/PPO ratio, concentration, and exposure duration may influence their interactions with biological systems. Pluronics, being biocompatible and having properties such as thermoreversible gelation, have found applications in orthopedics such as sustained drug delivery beneficial, for instance, for postsurgical pain management or promoting tissue healing;^23,24^ as scaffold materials in tissue engineering, where they provide mechanical support and a three-dimensional structure for bone or cartilage regeneration;^25–28^ and in synovial fluid mimicry as they possess lubricating properties that reduce friction and improve joint function.^29^ Based on the existing literature and identified research gaps, we hypothesize that (1) specific formulations of poloxamer-based hydrogels can be optimized to possess ideal rheological properties suitable for injectable applications in meniscus tear treatment; (2) these optimized formulations will demonstrate appropriate gelation temperatures and stability at body temperature required for in situ application; and (3) the selected poloxamer-based hydrogels will exhibit biocompatibility and support cell growth and migration necessary for meniscus tissue regeneration.

The presented article aims to evaluate various poloxamers and investigate the key properties and characterizations for further research toward injectable biomaterials for meniscus tear regeneration. The poloxamer-based hydrogels’ concentration and ability to undergo sol–gel transition in different temperatures and formulations were examined. Also rheological measurements were carried out to determine their exact gelation temperature and stability at body temperature. Finally, hydrogels were selected and eliminated in biological analysis, where an MTT assay was performed on mouse fibroblast cells to characterize the cell metabolic activity primarily. Furthermore, the chosen materials were observed in terms of cell growth and migration. Based on these findings, we selected the most promising poloxamer-based hydrogels for potential application as injectable materials for knee injuries.

## 2 Materials and Methods

### 2.1 Materials

Kolliphor K407 (PEO_200_PPO_65_PEO_200_), Poloxamer 188 Pro (PEO_153_PPO_29_PEO_153_), and Synperonic F108 (PEO_265_PPO_50_PEO_265_) were purchased from Merck Sigma-Aldrich (St. Louis, MO, USA). All chemicals were used as received. Ultrapurified water was obtained in-house using a Milli-Q water purification system (Millipore, Merck, Darmstadt, Germany). No significant hazards or risks or materials are associated with the reported work.

### 2.2 Preparation of Poloxamer-Based Hydrogels

Formulations composed of K407/P188 (KP), SF108/K407 (FK), and SF108/P188 (FP) in different combinations of 10%, 20%, or 30% w/w were prepared by mechanical dispersion in ultrapurified water at 4 °C and kept under refrigeration for 24 h. Table 1 shows the amounts of each component used and the concentrations tested. The formulations were stored at 4 °C for at least 24 h prior to further analysis.

**Table 1:** Specification of Name and Concentration of the Described Poloxamer-Based Hydrogels

Synperonic F108	Kolliphor 407	Kolliphor 407	Poloxamer 188	Synperonic F108	Poloxamer 188
F wt %	K wt %	K wt %	P wt %	F wt %	P wt %
30	30	30	30	30	30
30	20	30	20	30	20
30	10	30	10	30	10
20	30	20	30	20	30
20	20	20	20	20	20
20	10	20	10	20	10
10	30	10	30	10	30
10	20	10	20	10	20
10	10	10	10	10	10

### 2.3 Rheology

Rheological analysis of all formulations was performed at temperatures from 0 to 40 ± 0.1 °C using an MCR 702 rheometer (Anton Paar, RheoCompass) with parallel steel cone plate geometry PP25 L-LPP25/MD/ (60 mm, separated by a fixed distance of 0.095 mm). Samples were carefully placed on the inferior plate, ensuring the minimized sample shearing, and it was allowed to equilibrate for at least 15 min before analysis. The storage (elastic) modulus (G′) and the loss (viscous) modulus (G″) were measured following the temperature rise. The temperature variations were controlled during the sol–gel transition by a built-in temperature control chamber with an accuracy of ± 0.1 °C. At least three replicate samples were used to determine the rheological characteristics of each formulation.

#### 2.3.1 Sol–Gel Transition Temperature

The sol–gel temperature was assessed by rheological analysis, where the transition between elastic and viscous modulus occurred. Additionally, the gelation temperature was measured by using the tube inversion method. Briefly, poloxamer-based hydrogel (0.5 mL) was placed in 2 mL Eppendorf tubes equilibrated at 37 °C using a thermomixer. The samples were removed at regular intervals of 10 s and checked for gelation by inverting the tube. A digital timer was used to record the gelation time. Measurements were performed in triplicate for each of the formulations.

### 2.4 Biological Analysis—MTT Assay

Biological research was carried out under sterile conditions throughout the study. The measured hydrogels were prepared as described above. Cells were monitored under a microscope, and images were taken through the experiment to control the cell morphology. The test was performed based on the norm of ISO 10993-12:2012. Eppendorf tubes with hydrogels were placed in the incubator (37 °C, 5% CO_2_) for 10 min to allow gel phase transition and material stabilization. Subsequently, all samples were covered with 2 mL of DMEM/F-12K. The extraction process was conducted for 24 h at 37 °C. The pH of each extract was measured using the pH meter (Toledo). Each pH was reduced to 7.0–7.1 using sterile 0.1 M HCl. After that, the extracts were taken out and added to NIH/3T3 fibroblast cells. The general cytotoxicity assessment, using MTT, was performed.

### 2.5 Cell Migration—Scratch Assay

Cell migration was assessed by using the scratch assay. The NIH/3T3 cells in concentration of 3000 cells/well were seeded at the 96-well plate and left to grow for 24 h. After that time, a scratch was made across each well using a pipette tip. The wells were filled with hydrogel extracts prepared for the MTT assay in 24 h incubation of hydrogel with DMEM/F12K, and the pH was adjusted to 7.0 by sterile 0.1 M HCl solution. This scratch created an in vitro wound model, which was monitored by using an Olympus EP50 microscope at 48 and 72 h. The scratch width was measured, and images were taken until the wound closure. The cell migration was determined by using at least three replicate samples.

### 2.6 Cell Growth Kinetics

Real-time cell growth kinetics of NIH/3T3 cells were measured using an Agilent xCELLigence Real-Time Cell Analysis (RTCA) system. A sample of 150 μL of growth medium was added to each well of an Agilent E-Plate 96 to obtain background readings. For each well, 5,000 cells were seeded into the E-Plate 96 24 h before further analysis with extracts. The E-Plate containing cells was placed on the RTCA SP station in the cell culture incubator. Cell attachment, spreading, and proliferation were monitored every 30 min using the RTCA SP instrument. After 24 h, the measurement was paused to add hydrogel extracts, prepared as described in Section 2.5. Only 100% extracts were used for the experiment. Measurement was then continued for 7 days. The measured impedance recordings from cells in each individual well of the E-Plate 96 were automatically converted to Cell Index (CI) values by the RTCA software.

### 2.7 Statistical Analysis

All obtained results were subjected to statistical analysis using two-way ANOVA followed by Tukey’s multiple comparison test or Student’s *t*-test, where appropriate. Results are displayed as mean ± standard deviation. Sample size (*n*) is indicated within the corresponding figure legends. In all cases, significant differences were accepted when *p* < 0.05 with 95% CI, and GraphPad Prism 9 software was used throughout.

## 3 Results and Discussion

### 3.1 Preliminary Studies

To start, the preparation procedure of poloxamer-based hydrogels was interesting to observe and is worth noting. The preparation of poloxamer-based hydrogels is influenced by the ratio and molecular weight of PPO and PEO blocks as well as temperature, pH, and concentration. These elements affect the solubility, viscosity, and gel formation. Using the cold-method procedure, we observed that mixing at 4 °C overnight enabled gel formation after heating, while without mixing, gels did not form. This observation highlights the importance of temperature and mixing during dissolution.^30^ −.^31,32^ Consequently, all further materials were prepared via a cold-method procedure while mixing at 4 °C overnight. Preliminary tests excluded toxic materials and unsuitable gelation temperatures. Gelation stability was confirmed by inverting tubes at 37 °C (Figure 1a,b).

**Figure 1:**
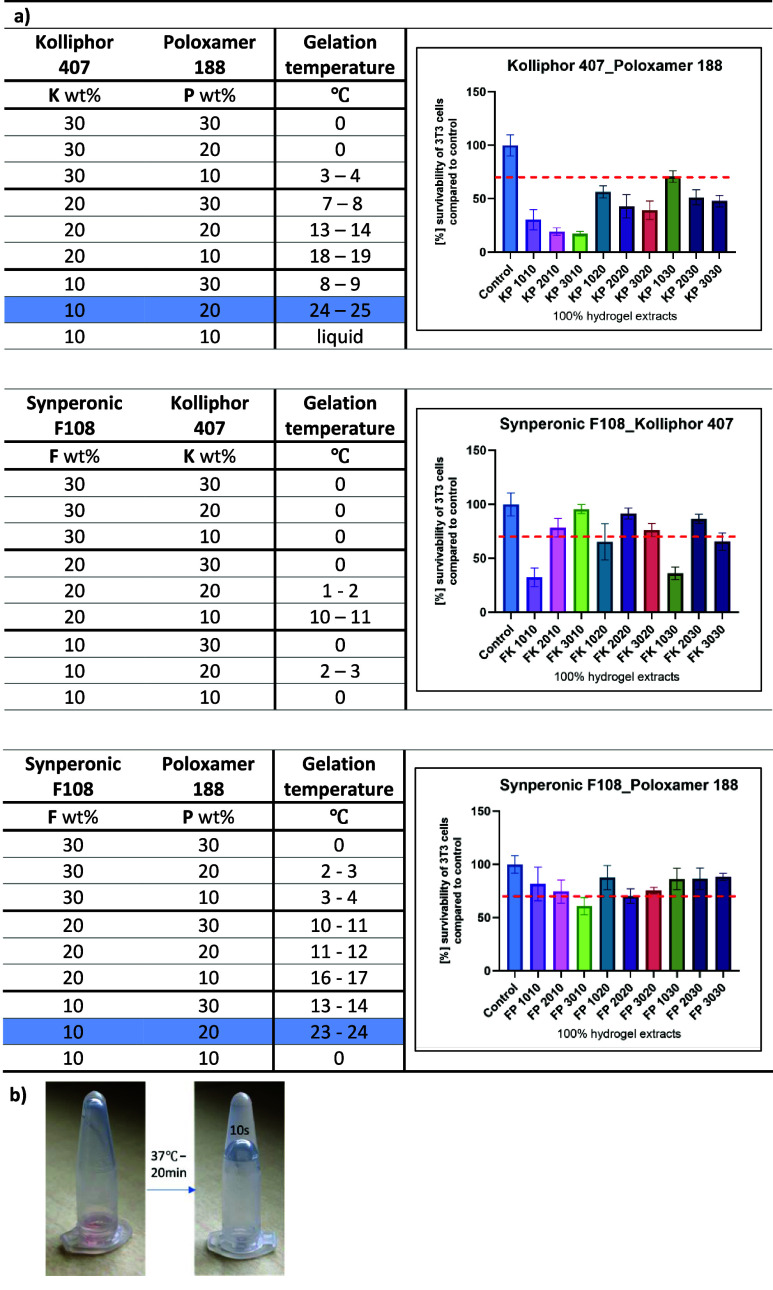
a) Data compilation of gelation temperatures obtained by the rheological measurements. The cytotoxicity graphs are for each group of materials. All error bars denote standard deviation, *n* = 4. The red line indicates 70% survivability according to the norm of ISO 10993. b) Representative Eppendorf tubes present the sol–gel transition of tested poloxamer-based hydrogels.

As we can see, the materials present a sol-to-gel transition with rising temperatures. We opted to select materials that gel close to body temperature. The hydrogels based on Synperonic F108 and Kolliphor 407 have not reached the gel stage even at temperatures above 40 °C. The materials based on Kolliphor 407 in higher concentrations presented the gel phase at lower temperatures. The optimal concentration for K407 was observed between 10% and 20%. Also, hydrogels consisting of Kolliphor 407 and Poloxamer 188 groups reached a gelling temperature above 20 °C, in contrast to Synperonic F108 with Kolliphor 407 hydrogels. This observation can be attributed to these surfactants’ different physical and chemical properties. Poloxamers are block copolymers that form thermoreversible gels, and their gelation behavior is highly dependent on the concentration and molecular weight of the individual components. Micellization and gel formation of poloxamers in the aqueous medium occur in two stages (Figure 2). During the initial stage, copolymers aggregate to form spherical micelles. The micelles’ outer layer comprises hydrated, swollen poly­(ethylene oxide) chains, while the dehydrated poly­(propylene oxide) blocks are found in the inner core. This first stage happens as the temperature rises and reaches the critical micellar temperature (CMT). Increasing the temperature leads to the overlapping of micelles and more organized alignment, resulting in the creation of gels. This process is reversible; thus, the gel returns to a viscous fluid state when the temperature is lowered to room temperature.^32–35^ It is known that the minimum gelation concentration decreases with increasing copolymer molar mass and PPO content and the critical micellar temperature decreases with increasing content of the more hydrophobic PPO block.^17,36–40^

**Figure 2:**
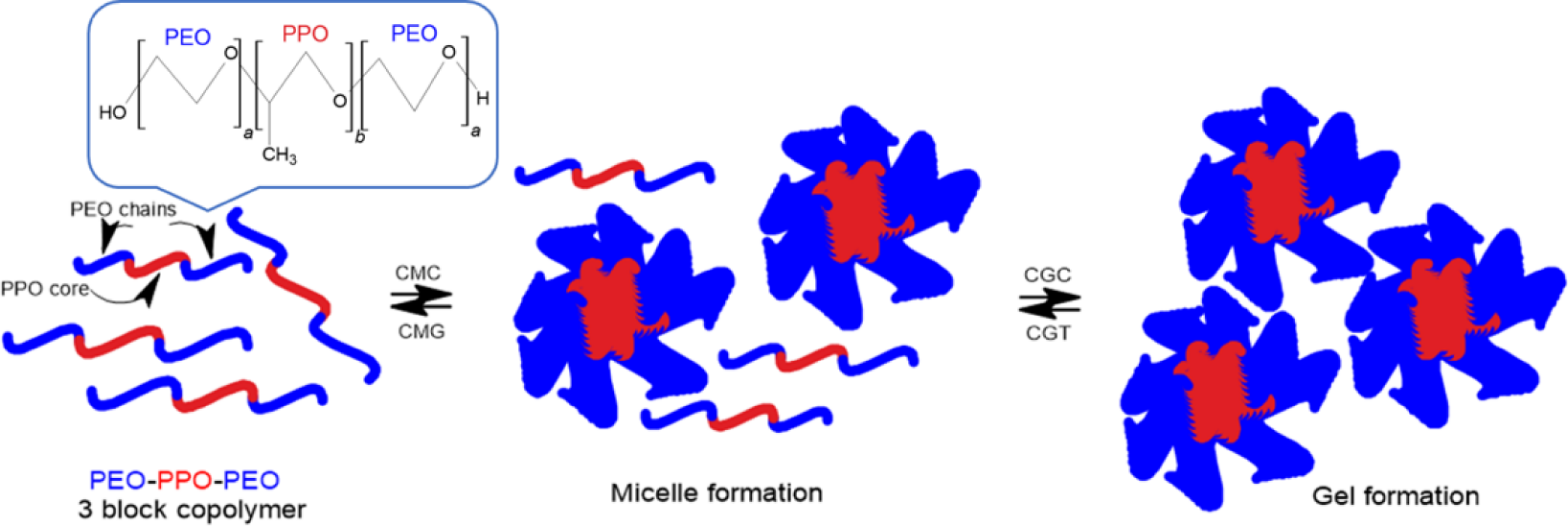
Schematic micellization and gel formation of poloxamer gels in an aqueous medium.

Poloxamer 188 (PEO_153_PPO_29_PEO_153_), for example, exhibits a gelation temperature that is often higher than that of Synperonic F108 (PEO_265_PPO_50_PEO_265_) due to its longer hydrophobic domains and higher molecular weight. Initial gelation experiments showed that FK hydrogels are unstable and have inappropriate gelling temperatures. Both Synperonic F108 (F) and Kolliphor P407 (K) contain PPO blocks of similar lengths, 50 and 56 units, respectively. The presence of these relatively long hydrophobic domains in both copolymers causes the systems composed of them to exhibit low gelation temperatures, significantly below body temperature. Due to the lack of desirable properties, FK hydrogels were no longer examined.^41–43^

A cytotoxicity test was conducted using hydrogel extracts in DMEM/F12K media, buffered initially, and supplemented with FBS and antibiotics. The pH of each poloxamer-based hydrogel was 7.0 before extraction and rose to 7.6/7.8 after incubation (Table 2). This pH shift is crucial, as slight changes can impact cell viability and morphology, affecting MTT test results. Similar pH increases were reported in studies of thermoresponsive polymers, where pH changes affect protein structure, enzymatic activity, and cellular function.^44–47^ Poloxamer breakdown products, including ethylene glycol and fatty acids, may cause this shift, altering ionic composition, osmotic pressure, and pH.^44,48–51^

**Table 2:** pH Comparison of Poloxamer-Based Hydrogels after Preparation and pH of the Extraction Medium after Hydrogel Incubation with the Complete Medium

Kolliphor 407	Poloxamer 188	Clear solution	Extraction medium	Synperonic F108	Poloxamer 188	Clear solution	Extraction medium
K wt %	P wt %	pH	pH	F wt %	P wt %	pH	pH
30	30	7.1	7.8	30	30	7.1	7.6
30	20	7.0	7.6	30	20	7.0	7.6
30	10	7.1	7.8	30	10	7.0	7.5
20	30	7.0	7.6	20	30	7.0	7.5
20	20	7.0	7.6	20	20	7.0	7.6
20	10	7.0	7.6	20	10	7.0	7.6
10	30	7.0	7.4	10	30	7.0	7.4
10	20	7.0	7.6	10	20	7.0	7.5
10	10	7.0	7.5	10	10	7.0	7.6

KP gels presented lower biocompatibility compared with the other hydrogels tested. This may be related to the interactions between the hydrogel components and the cells. Kolliphor 407, known for its surfactant-like properties, can alter the cell membrane integrity, reducing biocompatibility. The highest biocompatibility for all tested concentrations was obtained for hydrogels based on Synperonic F108 and Poloxamer 188. Considering their surfactant nature, the toxicity decreases as the molecular weight of the PPO part increases, and a large hydrophilic crown is formed that well hides a hydrophobic core of micelles.^41^ Nevertheless, the materials performed with higher cytotoxicity than expected. The increased pH may be one of the contributing factors to cytotoxicity in the MTT assay. It is probably due to the method used to determine the metabolic activity of NIH/3T3 cells, and the extracted medium was used to perform the MTT test. It has been observed that after 24 h of hydrogel incubation with cell culture medium, the pH of the extracted medium was increased from neutral to pH 7.6, significantly affecting the viability of fibroblast cells (Figure 3a,b). Thus, a pH adjustment must have been made to exclude the pH negative effect. After 24 h incubation, the extracts were filtered by a 0.22 μm PES membrane filter, and then the pH adjustment was made using the sterile 0.1 M HCl solution. After that, the MTT assay was proceeded, giving efficient results of the cytotoxic effect of basal concentrations of each poloxamer hydrogel component (Figure 3c).

**Figure 3:**
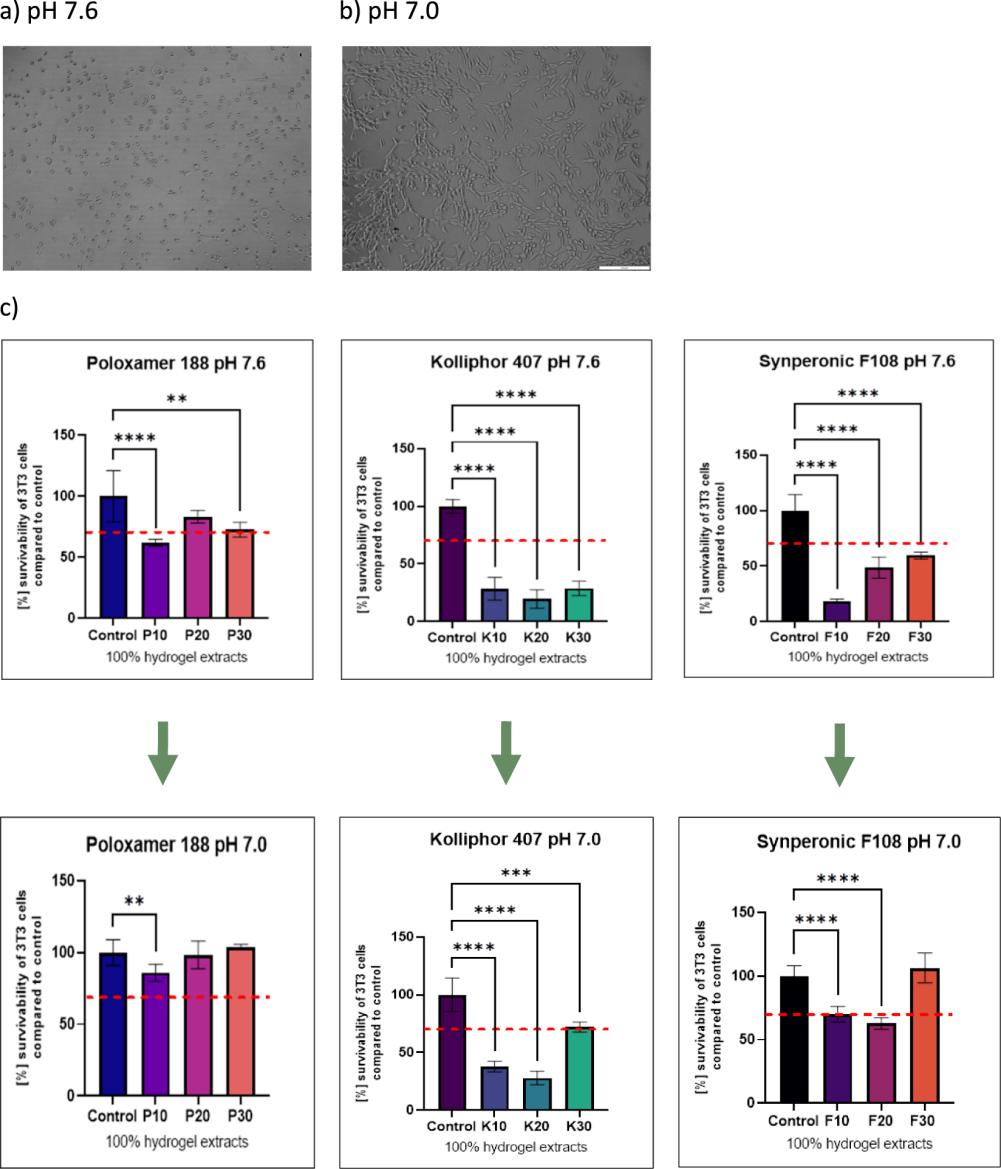
An image of a cell culture well after 24 h incubation with material extract of a) pH 7.6 and b) pH 7.0. Scale bar is equal to 200 μm. c) An in vitro cytotoxicity test of the hydrogels by MTT assay, performed after the pH adjustment for the material extracts after 24 h incubation in DMEM/F12K. Statistical significance: *Compared to the control group; **p < 0.01; ***p < 0.001; ****p < 0.0001. The red line indicates 70% survivability according to the norm of ISO 10993. All error bars denote standard deviation, *n* = 4.

As presented, the highest cytotoxic effect is observed for Kolliphor 407 in lower concentrations, where the ratio of Poloxamer 188 and Synperonic F108 significantly increased and no toxic effect was observed.

To continue with the experiments, based on so far obtained results, we added one more material concentration, the KP 1515 and FP 1515, due to the presumably optimal gelling temperatures near 20%, bearing in mind the influence of the Poloxamer 188 to decrease the sol–gel temperature while combined with K407 or SF108.

### 3.2 Rheology

We then continued the experimental part by measuring the sol–gel transition of poloxamer-based material in the storage modulus (G′) and loss modulus (G″) functions as viscosity changes with rising temperature. Up to this point, we had selected six different poloxamer-based materials that exhibited the closest gelling temperature and biocompatibility. The results obtained for a representative group of hydrogels are presented in Figure 4.

**Figure 4:**
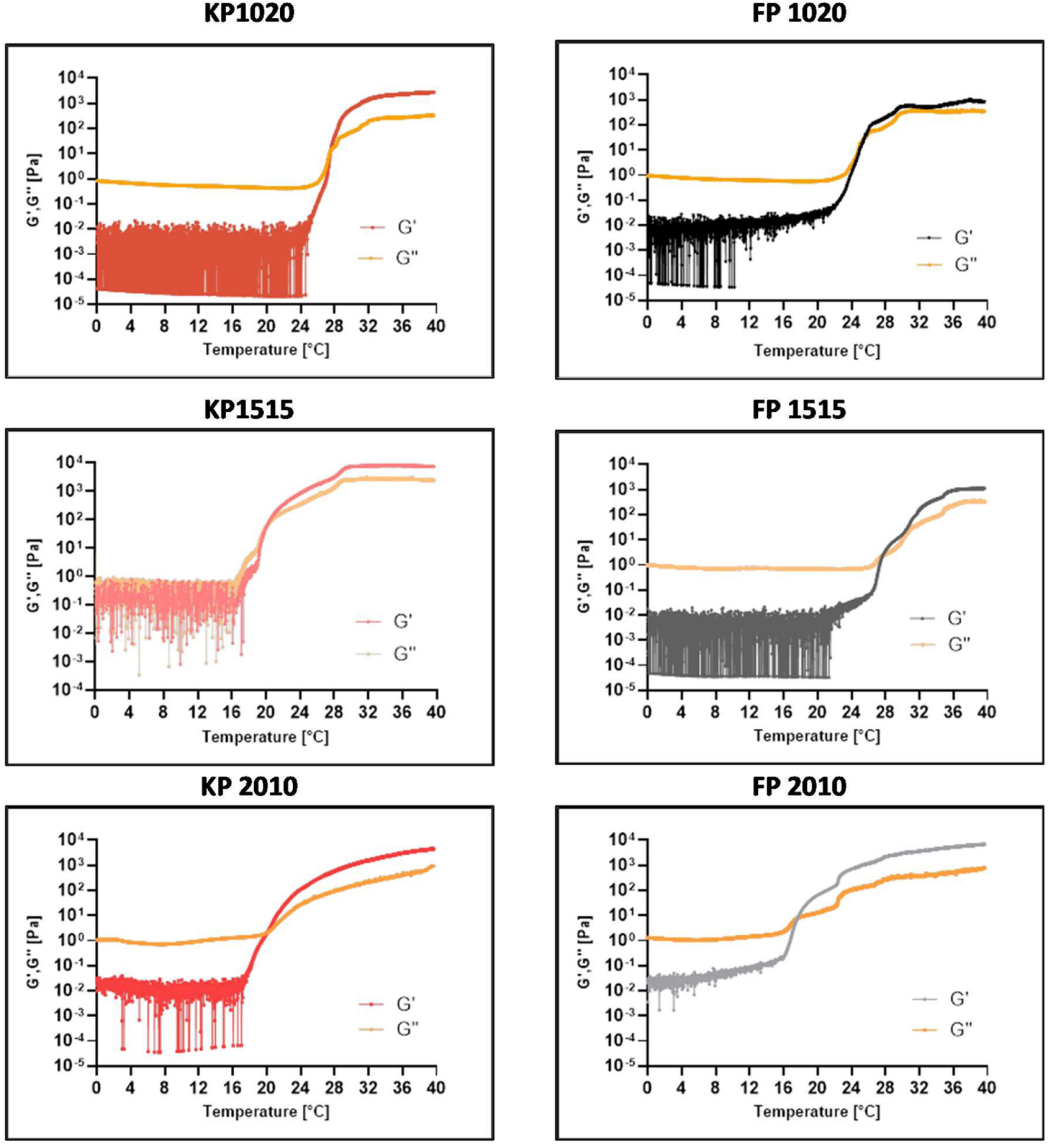
Representative rheology results of the storage modulus (G′) and loss modulus (G″) as viscosity changes with rising temperature on poloxamer-based hydrogels.

As we can see, all materials exhibit gelling temperatures from 16 to 28 °C. Higher temperatures were recorded for materials with concentrations of Kolliphor 407 and Synperonic F108 lower than 20 wt %. This result aligns with previous studies, which have shown that the higher concentration of amphiphilic block copolymers like Poloxamer 407 can facilitate gel formation at lower temperatures, making it more suitable for biomedical applications that require gelation at or near body temperature.^1,53^ Poloxamer 188 resulted in a perfect bonding copolymer for reducing or increasing the gelling temperature with higher concentrations.

### 3.3 Cytotoxicity Test by MTT Assay for Selected Pluronic-Based Hydrogels

Based on the results, we focused on poloxamer-based hydrogels that met our criteria of biocompatibility and sol–gel transition near body temperature. We selected hydrogels with ≤ 30 wt % poloxamer concentration, dividing them into two groups: Group 1 (KP 1020, KP 1515) for injectable applications, and Group 2 (FP 1020, FP 1515) for lubricable hydrogel use. Following that, we performed cell culture studies starting with an MTT assay, which prolonged the incubation time to 72 h and 7 days (Figure 5).

**Figure 5:**
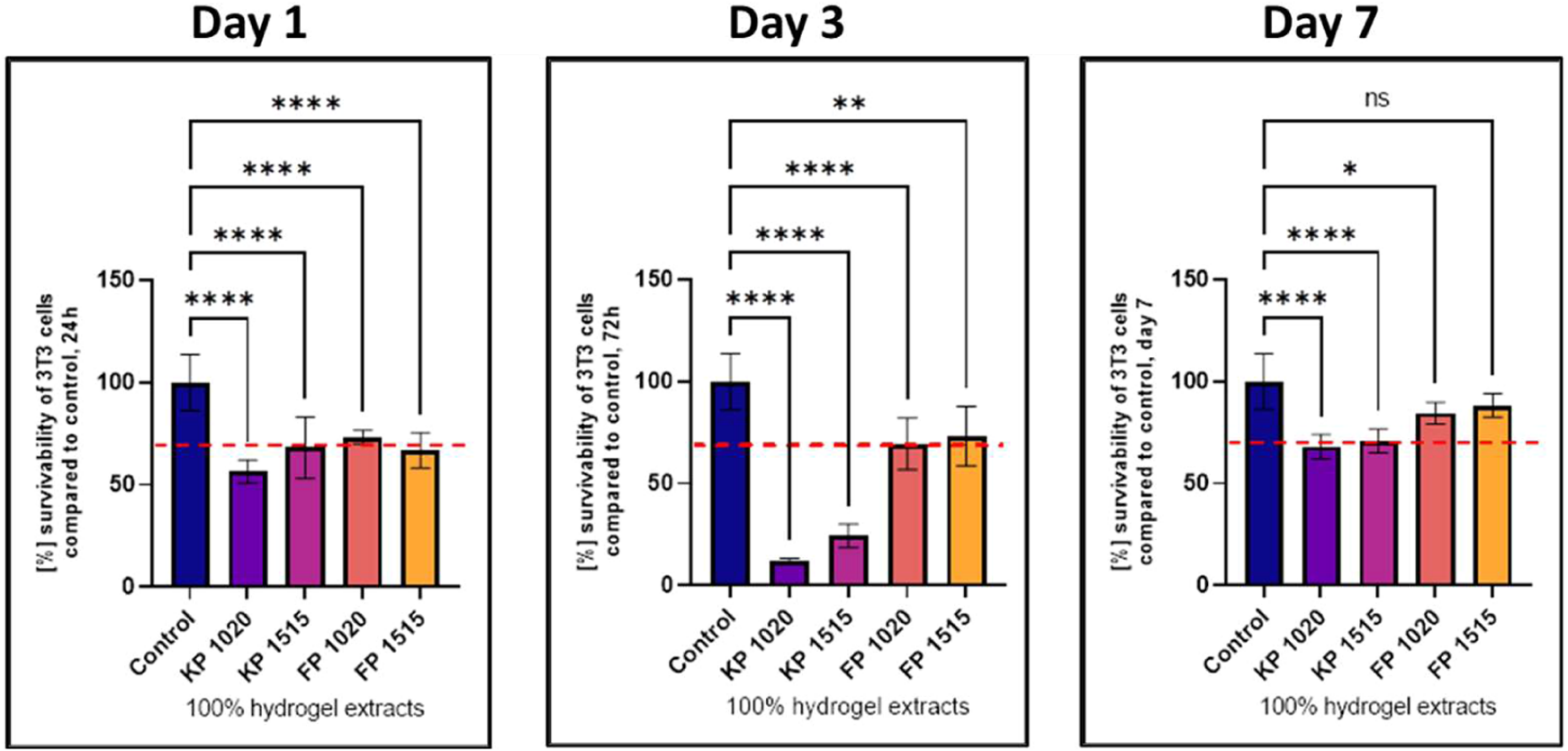
Cytotoxicity results of selected hydrogels performed using the MTT assay after 24, 72, and 7 days. Statistical significance: *Compared to the control group; ****p < 0.0001, all error bars denote standard deviation, *n* = 4. The red line indicates 70% survivability according to the norm of ISO 10993.

By day 7, three formulations (FP1515, FP1020, and KP1515) exhibited excellent biocompatibility with viability values exceeding 90% relative to the control, with no statistically significant differences between them. The dynamic viability pattern of KP1020, showing an initial decline followed by recovery, suggests potential cellular adaptation mechanisms that warrant further investigation. The consistent performance of FP1515 across all time points makes it particularly promising for applications requiring stable biocompatibility from the early stages of implantation.^46,54^

These findings align with studies that report the long-term benefits of poloxamer-based hydrogels in promoting cell proliferation and tissue regeneration.^55–57^ The biocompatibility of these hydrogels is crucial for applications such as drug delivery, where prolonged cell contact and in situ gelation are needed. Their ability to support cell viability while avoiding cytotoxicity makes them ideal for injectable hydrogel formulations requiring sustained cell interactions.

### 3.4 Cell Growth Kinetics

These four hydrogels were further tested toward dynamic cell growth kinetics, where the extracted medium was added after 24 h and monitored for 7 days (Figure 6).

**Figure 6:**
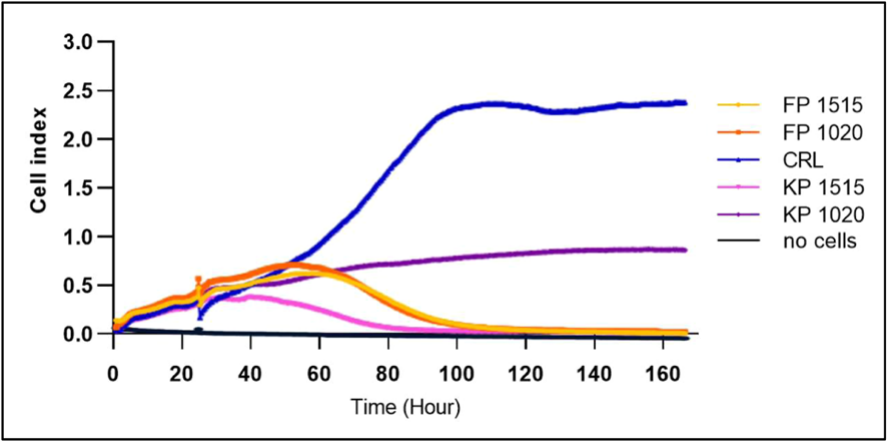
Dynamic proliferation curve of 3T3 fibroblast cells. Cells were seeded in the Agilent E-Plate 96 and continuously monitored every 30 min using the Agilent RTCA SP instrument.

The dynamic cell proliferation was different depending on the extracted medium, which was previously adjusted to pH 7.0 as described above, and only 100% extracts were added to each well after 24 h of cell incubation to allow cells to attach. Our RTCA data clearly show that all hydrogel formulations resulted in reduced cell proliferation compared to the control (CRL). Contrary to our initial interpretation based on the MTT assay, none of the tested formulations maintained proliferation comparable to that of the control throughout the measurement period. KP1020, which we previously highlighted for its favorable performance in the MTT assay, shows a significant proliferation decline after day 3. Similarly, FP1515 and FP1020 exhibit initial maintenance of cell index values, followed by a gradual decline after day 2. KP1515 demonstrated the least inhibitory effect among all tested formulations during the first 48 h. The lower cell proliferation observed for both FP hydrogels might be due to the cell-prone aggregation, which caused a more rapid cell reduction but did not reach the black line. Poloxamer 188 in concentration 20 wt % is optimal for cell viability enhancers.^42,43^ This discrepancy between our MTT results (Figure 5) and RTCA proliferation data suggests that while our formulations may support cell survival, they do not promote robust proliferation to the same extent as the control medium. These changes may be due to the lack of availability of fresh medium after the third day of incubation. This is because 100% of the extracts were added to the wells, which were hydrogels diffused into the cell culture medium.

### 3.5 Cell Migration—Scratch Assay

Proceeding with the selected four materials, due to satisfactory sol–gel temperature change, cell viability, low toxicity, and proliferation speed in direct cell-hydrogel contact, the final cell culture test was performed, where a scratch assay was used, and the wound closure was photographed up to day 7. The results are presented in Figure 7.

**Figure 7:**
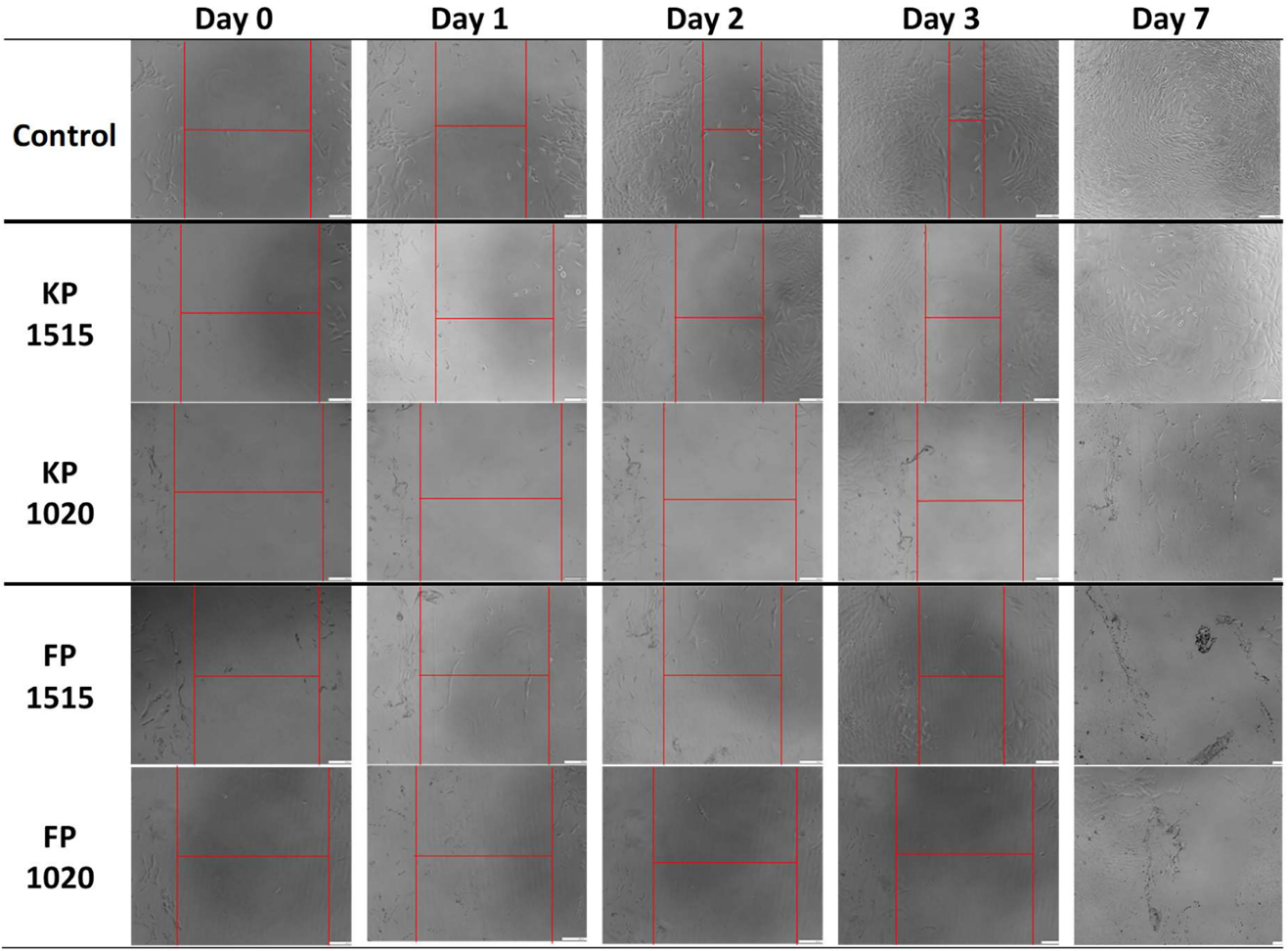
Scratch test images with determined gap closure from day 0 to day 3 of each hydrogel sample compared to the control. All scale bars are 200 μm.

As we can see, the NIH/3T3 migration in concentration of 3000 cells/well begins within 3 days, whereas in the control group, the “scratch” already starts to close significantly earlier. Following that, all of the materials based on KP showed the slow closure of the created wound. The created gap was closed by the fifth day of the test in the control and KP groups, whereas the complete closure for the FP groups was observed after 7–8 days. Qualitative analysis of the wound closure images clearly shows a time-dependent difference between the groups, with control samples exhibiting the fastest closure rate, followed by KP formulations, while FP formulations demonstrated markedly delayed wound healing. Wells covered by the FP materials presented a less visible gap closure, which suggested slower cell migration and wound healing capacity in this environment.

However, it is worth noting that the FP hydrogels prompted more cell aggregation that evenly spread the cell distribution within the well. In contrast, the hydrogels based on KP were more cell adhesive and opted for even cell spreading. This suggests that both materials are biocompatible but possess slightly different properties when in direct contact with the NIH/3T3 cells. It is important to note that these migration findings are consistent with our RTCA proliferation data (Figure 7), which also indicated limited cellular activity in the presence of FP hydrogels compared to control conditions.

This slower migration in FP-based hydrogels might be due to several factors. First, Kolliphor 407 hydrogels promote more adhesive interactions between the hydrogel matrix and fibroblast cells, which could lead to localized cell attachment and hinder the efficient movement of cells across the wound site.^58–61^ Conversely, Poloxamer 188 hydrogels led to more uniform cell aggregation, which might be due to the different gelation characteristics of Poloxamer 188, promoting even cell distribution throughout the hydrogel matrix. This observation supports the hypothesis that cell aggregation in FP hydrogels may promote a more dispersed distribution pattern, leading to slower migration but even cell distribution. This aligns with findings in the literature, which demonstrate that both Kolliphor 407 and Poloxamer 188 are biocompatible and promote cell survival and proliferation in various in vitro and in vivo models.^2,42,62,63^ These results are significant because biocompatibility is crucial for the safety of hydrogels in medical applications, particularly in drug delivery, wound healing, and tissue engineering.

## 4 Conclusion

In conclusion, we successfully developed and characterized poloxamer-based hydrogels by blending Poloxamer 188, Kolliphor 407, and Synperonic F108 in varying concentrations. Through rheological studies, we identified four formulations with optimal sol–gel transitions near body temperature: KP 1020, KP 1515, FP 1020, and FP 1515.

Our cytotoxicity studies revealed that higher concentrations of Poloxamer 188 enhanced the metabolic activity of NIH/3T3 cells, aligning with literature research demonstrating P188’s role in cartilage production and chondrocyte protection.^2,42,64^ An important observation was the pH change when in contact with the cell culture medium, which needs to be further examined. This characteristic suggests potential applications in pH-responsive drug delivery systems, particularly when combined with thermoresponsive properties. Such dual-responsive systems are increasingly relevant for targeted drug delivery applications.^52,65,66^ Despite promising results, our study has some limitations, which include the use of a single cell line (NIH/3T3) for biocompatibility tests, the lack of in vivo studies, and the absence of mechanical testing under cyclic loading, which is essential for knee joint applications.

Compared with existing commercial alternatives, our hydrogels offer several potential advantages. Currently used knee joint visco-supplementation preparations, such as hyaluronic acid (e.g., Synvisc, Euflexxa), show limited durability in the joint (14–30 days) and mainly act as lubricants without a significant impact on tissue regeneration. Our hydrogels, particularly the KP1020 and FP1515 formulations, potentially offer a longer retention time due to in situ sol–gel transition and the possibility of easy incorporation of bioactive factors. Compared to available collagen scaffolds used in meniscus surgery (e.g., CMI, Menaflex), injectable hydrogels enable minimally invasive administration, which significantly reduces surgical trauma and shortens recovery time. Furthermore, unlike animal-derived products, poloxamer hydrogels minimize the risk of immunological reactions and pathogen transmission. Our hydrogels also show potential for modification of their mechanical properties over a wider range than commercially available synthetic substitutes, allowing for better customization to the specific needs of the patient. Also, unlike the already existing scaffolds (ACTIfit or Biocartilage), which are scaffolds for over a microfracture defect, there is a limitation to only partial defected coverage. Meanwhile, our materials fill the damage inside, covering the wound and protecting it. When mixed with active factors, they penetrate and induce healing.

Despite promising results, clinical translation faces challenges, including sterility, long-term stability, and in vivo assessments. Future research should optimize the mechanical properties and explore bioactive molecule incorporation for enhanced healing. These developments could significantly advance the field of regenerative medicine, particularly in osteochondral repair and tissue engineering applications.

## Data Availability

The data presented in this study are available on request from the corresponding author.
